# Diel niche partitioning of a plant-hummingbird network in the Atlantic forest of Brazil

**DOI:** 10.1007/s00442-023-05347-4

**Published:** 2023-04-07

**Authors:** Andrea Nieto, Rafael O. Wüest, Catherine H. Graham, Isabela G. Varassin

**Affiliations:** 1 Laboratório de Interações e Biologia Reprodutiva, Universidade Federal do Paraná, Centro Politécnico, Jardim das Américas, 81531-990 Curitiba, PR Brazil; 2 Programa de Pós-Graduação em Ecologia e Conservação, Universidade Federal do Paraná, Centro Politécnico, Jardim das Américas, 81531-990 Curitiba, PR Brazil; 3 Swiss Federal Institute for Forest, Snow and Landscape Research WSL, Zürcherstrasse 111, 8903 Birmensdorf, Switzerland; 4 Senckenberg Biodiversity and Climate Research Center (SBiK-F), Senckenberganlage 25, 60325 Frankfurt, Germany

**Keywords:** Competition, Co-flowering, Facilitation, Interaction, Nectar

## Abstract

Niche partitioning is an important mechanism that allows species to coexist. Within mutualistic interaction networks, diel niche partitioning, i.e., partitioning of resources throughout the day, has been neglected. We explored diel niche partitioning of a plant-hummingbird network in the Brazilian Atlantic forest for nine months. To evaluate diel patterns of hummingbird visits and nectar production, we used time-lapse cameras on focal flowers and repeated nectar volume and concentration measures, respectively. Additionally, we measured flower abundance around focal flowers and flower morphological traits. We did not observe diel partitioning for either hummingbirds or plants. Instead, hummingbirds appeared to specialize in different plant species, consistent with trophic niche partitioning, potentially resulting from competition. In contrast, plant species that co-flowered and shared hummingbird visits produced nectar during similar times, consistent with facilitation. Our focus on the fine-scale temporal pattern revealed that plants and hummingbirds appear to have different strategies for promoting co-existence.

## Introduction

Niche partitioning is an important mechanism that allows similar species to coexist (Schoener 1974; Chesson 2000). Ecological theory predicts that species in the same guild may compete for limited resources (Levine and HilleRisLambers 2009) but sufficient differences in their niche (i.e., divergence in resource utilization) through morphology, physiology, or behavior may allow coexistence (Tilman 1987). Such a mechanism minimizes competition among species and contributes to the maintenance of diversity, especially in species-rich areas such as the tropics.

Competing species may partition their niche through diet (hereafter termed “trophic niche partitioning,” Maglianesi et al. 2015), space (Lara et al. 2011), or time (Aguilar-Rodríguez et al. 2019). Within plant-pollinator systems, specialized morphologies may promote trophic niche partitioning, leading, for instance, long-billed hummingbirds to prefer long flowers even when short flowers are available (Weinstein and Graham 2017). However, when flower abundance decreases and similar pollinators are limited to visiting the same floral resources, diel niche partitioning could be an alternative to avoid competition (Kronfeld-Schor and Dayan 2003). For example, when a single plant species provided the main nectar resource in a pine-oak forest in Mexico, large species of hummingbirds foraged in the morning while smaller ones foraged in the afternoon (Lara et al. 2009). In a similar case, large-aggressive hummingbirds dominated the floral visits over the smaller ones during the most nectar-limited time of an agave species in Mexico, suggesting a diel partitioning of floral rewards among pollinators (Ornelas et al. 2002). Although diel niche partitioning has rarely been observed among pollinators, it could be an important mechanism for structuring the pollinator-plant system.

Diel niche partitioning may be an effective mechanism when sharing similar resources. For instance, sympatric plant species that are unable to diverge in space, flowering season (hereafter termed “co-flowering”) and/or pollinator guild (Stone et al. 1998) are likely to share pollinator services. In such cases, plants may release pollen or nectar at different times throughout the day to partition the activity of shared pollinators (hereafter termed “pollinator niche”). Different times of resource release from plant species can reduce competition for pollinator visits (Armbruster and Herzig 1984; Stone et al. 1998). While some studies have explored diel niche partitioning in plants, these are generally focused on a single species or guild and its pollinators (Armbruster and Herzig 1984; Stone et al. 1998), neglecting the community context, which could be particularly important in diverse communities. Understanding diel niche partitioning is essential, as it may explain the high biodiversity in rich communities of mutualistic species such as plants and pollinators.

Although competition may lead to niche partitioning and explain coexistence, some studies suggest that plant coexistence might be driven by facilitation (Tur et al. 2016; Bergamo et al. 2020a, b). For instance, rare species could benefit from the presence of abundant species when their visitation rates increase (Tur et al. 2016; Wei et al. 2021) and this kind of asymmetric facilitation has been shown to foster plant-pollinator coexistence in biodiverse systems (Bergamo et al 2020a; Wei et al. 2021). Facilitative interactions might occur due to similarity in phenology, flower traits, and nectar content (Bergamo et al. 2018) leading to joint attraction of pollinators to rare and abundant species in a given community (Bergamo et al. 2020a). It is, therefore, important to consider studying both positive and negative interactions within plant-pollinator systems since the balance between competition and facilitation in plant-pollinator interactions is context-dependent (Benadi and Paw 2018; Bergamo et al 2020b).

Plant-hummingbird interactions are well suited to explore diel niche partitioning. In a diverse ecosystem, such as the Atlantic forest (Myers et al. 2000), many hummingbird-pollinated plants (e.g., bromeliads—see Buzato et al. 2000) co-flower and present flowers that last only one day (e.g., Martinelli 1995). In this scenario, timing is crucial for hummingbirds interacting with each other to feed on the same ephemeral flowers (Feinsinger and Colwell 1978) and for plants relying on floral visitors for pollen transportation (Morales and Traveset 2008; Ashman and Arceo-Gómez 2013; Arceo-Gómez and Ashman 2016). Here we explore diel niche partitioning from plant and hummingbird perspectives in a diverse tropical region, the Atlantic forest. We collected data on hummingbird floral visitation and nectar production from plants visited by hummingbirds. We hypothesized that if competition structures this mutualistic network, we expect a trophic or diel niche partitioning among plants or pollinators. Specifically, we expected hummingbirds to show either (I) trophic niche partitioning, i.e., hummingbirds partition floral resources by foraging on a subset of different flowers, or (II) diel niche partitioning, i.e., hummingbirds partition floral resources by foraging at different times throughout the day. We expected plants to show a diel partitioning of the pollinator niche when plants (III) co-flowered, (IV) shared a hummingbird species, or (V) were morphologically similar in floral traits associated with pollen placement.

## Methods

### Study site and sampling design

Our study was conducted within the Estação Biológica Santa Lúcia (EBSL, 19°59′S, 40°32′W), southeastern Brazil. The mean annual temperature is 19 °C and varies from 14.3 to 26.2 °C (Thomaz and Monteiro 1997). The average annual precipitation is 1900 mm, with the highest rainfall in November and the lowest in June (Mendes and Padovan 2000). The study area is old-growth mainly tropical rainforest (Atlantic forest) (Mendes and Padovan 2000), where the understory tends to be dominated by bromeliads (Wendt et al. 2010). Within EBSL, we established a 1.5 km by 10 m transect where we collected data on plant-hummingbird interactions, flower abundance and traits, and nectar production. We collected data once a month between Nov-2018 and Jul-2019.

### Plant-hummingbird interaction sampling

We counted plant-hummingbird visits using time-lapse cameras (Plotwatcher Pro—12 cameras) distributed along the transect. This method minimizes the time spent obtaining plant-hummingbird interaction resulting in increased data collection in time and space (Weinstein 2015; Weinstein and Graham 2017). We placed a single camera (up to 2 m above the ground) for three days at a flower or group of flowers for 12 flowering plants on the transect each month. We distributed the cameras to maximize the number of plant species each month. The cameras took an image every second from dawn to dusk (~ 12 h), generating ~ 43,200 images per camera per day. We found frames with hummingbirds using Deep Meerkat software (Weinstein 2015). We selected frames with legitimate visits only (i.e., in which the birds inserted their bills into the corolla tube) and identified the hummingbird species. We set a time interval of 20 s among visits to define independent visits.

### Flower abundance and trait sampling

To estimate flower abundance, we counted all open flowers (visited or not by hummingbirds) fitting the traditional ornithophilous syndrome, i.e., red to purple and long corolla tubes (Fægri and van der Pijl 1972) along the transect. When possible, we counted all open flowers on a plant. When we found a dense flowering plant, we counted flowers on five branches (or inflorescences), calculated the average, and then multiplied the average by the total number of branches (or inflorescences). Given that flowers with bat or insect pollination syndromes are likely to be visited by hummingbirds (Dalsgaard et al. 2009), we placed cameras on these species and only excluded them when visits were not recorded.

To assess whether plant species visited by hummingbirds produced nectar at different times throughout the day, we extracted nectar at sequential time slots, from 06:00 to 18:00 h every four hours. For each plant species, we bagged flower buds. Once the flowers opened, we collected the nectar of two flowers of five individuals in each time slot (n total = 40 flowers). We sampled flowers from less than five individuals when we did not find enough. We avoided measuring nectar in previously damaged flowers (Kearns and Inouye 1993). We extracted nectar using a microliter syringe (Hamilton syringe 50 µl) or capillary tubes (20 and 60 µl); both methods allowed us to collect the total reward offered by the flower. We measured the total sugar content in nectar using a manual refractometer (concentration range 0–32% Brix scale = weight of sugar per volume of solution at a given temperature, Kearns and Inouye 1993). We corrected the Brix values because they are temperature-dependent (Cruden et al. 1983).

We applied the cumulative nectar method (Gill 1988; Heil 2011), which consists of extracting nectar from “newly” opened flowers (i.e., those that have just opened their petals for the first time) in each time slot. For plant species whose flower abundance was low (*Aechmea lamarchei*, *Billbergia amoena*, and *Nidularium procerum*) we could not use cumulative nectar method. Instead, we applied the dynamic method (Heil 2011) by repeatedly extracting nectar from the same flowers in each time slot, i.e., the individual flowers used to extract nectar at 06:00 were the same to extract nectar at 10:00, 14:00 and 18:00 h. To make the nectar production obtained with the cumulative and dynamic methods comparable, we corrected the Brix values of the latter method as follows: for extractions at 06:00 h, we used the volume and concentration values observed in this time slot. For subsequent extractions, we added the value of the volume obtained in the previous time slot and kept the concentration value because we expected a slight variation of nectar concentration within the same plant species (Varassin et al. 2001; McDade and Weeks 2004). When the flower had zero nectar, we used the volume and concentration values observed in the previous time slot, i.e., we used the volume and concentration recorded at 06:00 h for a flower that had zero nectar at 10:00 h. Once standardized both cumulative and dynamic methods, we calculated nectar production (mg sugar) as the product of nectar volume (ml) by sugar concentration (mg ml^−1^), following Kearns and Inouye (1993), although non-sugar constituents may also influence the sugar concentration (Inouye et al. 1980).

To examine floral morphology, we measured anther height as the distance from the base of the corolla tube to the tip of the anther. We selected anther height because it can influence the location of pollen placement on the body of pollinators (Rocca and Sazima 2013; Fonseca et al. 2016; Bergamo et al. 2017), which could result in niche partitioning among plant species. We collected at least five flowers, each one from a different individual of each plant species visited by hummingbirds. We took a scaled photograph for each flower and measured it using ImageJ software (Schneider et al. 2012). We collected one individual of each plant species as a voucher specimen that was deposited at the MBML herbarium—Instituto Nacional da Mata Atlântica. Plant species were identified by comparisons with plant species vouchers by local specialists (see Acknowledgements).

## Data analysis

### Hummingbirds niche partitioning

**Fig. 1:**
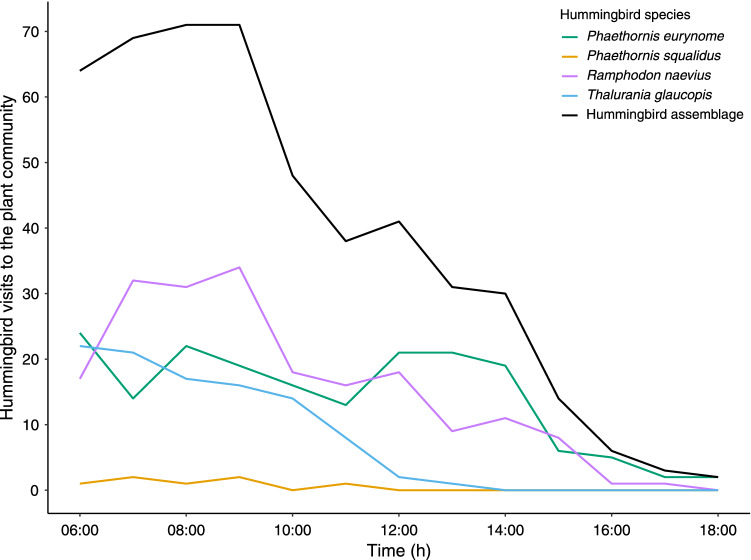
Number of hummingbird visits to the plant community throughout the day at EBSL, southeastern Brazil. Each line represents the pattern of visits of each hummingbird species to the plant community

We analyzed trophic (following Winemiller and Pianka 1990) and diel niche partitioning (following Castro-Arellano et al. 2010) among hummingbirds. For the trophic niche partitioning, we built a matrix M1 populated with the observed interactions between hummingbird (N rows) and plant species (M columns). For the diel niche partitioning, we built a second matrix M2 populated with the observed interactions for each hummingbird species (N rows) throughout the hours of a day (from 06:00 to 18:00 h, 12 columns). Since most hummingbird visits occurred in the morning (Fig. 1), we also tested the diel niche partitioning considering only the morning (from 06:00 to 12:00 h). We quantified trophic niche partitioning from M1 (hummingbird-plant interactions) and diel niche partitioning from M2 (diel hummingbird activity) by calculating the mean of the Pianka index derived from all possible pairwise combinations of the hummingbird species (Pianka 1973). Adapted to our question, the index ranges from 0, for complete partitioning (hummingbirds foraging on different plant species for trophic niche partitioning, or during different times for diel niche partitioning), to 1, for no partitioning (hummingbirds foraging on the same plant species or at the same times).

We used two different null models, one for trophic niche partitioning (‘ra3’ following Albrecht and Gotelli 2001) and another for diel niche partitioning (‘ROSARIO’ following Castro-Arellano et al. 2010), to test if the observed niche partitioning deviates from what can be expected by chance (i.e., when hummingbirds randomly selected plants to interact with or randomly interact throughout the day or morning). For trophic niche partitioning, we created 10,000 null distributions by shuffling the entries in each row of the matrix (‘ra3’). This ensures that the niche breadth of hummingbirds (the number of plants they interact with) is kept constant while allowing the utilization of any possible interaction partner. For diel niche partitioning, we created 10,000 null distributions by randomizing the daily activity patterns (rows in M2) over a time domain. In each randomization (‘ROSARIO’ algorithm), the activity patterns of the hummingbird species advance in sequence occupying a new location in the time domain. ROSARIO considers time as a sequential, continuous, and ordered resource and maintains temporal autocorrelation of data by shifting only the distribution and not the shape of the activity patterns of the species throughout the day (e.g., the pattern of hummingbird visits observed at 06:00 is moved to 07:00 h, the pattern observed at 08:00 is moved to 09:00 h, and so on) (Castro-Arellano et al. 2010). For both trophic and diel niche partitioning, observed values lower than random indicated segregated activity patterns (i.e., niche partitioning). In contrast, observed values higher than random indicated coincident activity patterns (i.e., niche overlap, Castro-Arellano et al. 2010). We simulated trophic niche partitioning by using the niche.overlap function of the EcoSimR package (Gotelli et al. 2015) in R programming language (R Core Team 2018) and, the diel niche partitioning by using the Time Overlap software (Castro-Arellano et al. 2010).

### Plants niche partitioning

We followed the above-described approach to evaluate diel partitioning of the pollinator niche among plants (‘ROSARIO’, Castro-Arellano et al. 2010). This required to come up with a third matrix M3 that contains the availability of resources for each plant species (M rows) throughout the hours of a day (from 06:00 to 18:00 h, 12 columns). We populated this matrix by estimating nectar production using a generalized linear model (GLM) with the Poisson distribution because we did not have nectar data for every hour of the day. The Poisson GLM used a linear and a quadratic term to relate hours to nectar production (sugar content in µg rounded to integers) for each plant species. We then used the fitted models to interpolate species-specific values of µg sugar at each hour (from 06:00 to 18:00 h). We estimated the total availability of resources for each plant species by multiplying the nectar production (mg sugar) by the total number of flowers, which was the total sum of flowers during the sampling period for each plant species.

Analogously to the hummingbird case, we calculated the diel niche partitioning of the community of plants among all possible pairwise combinations of plant species using the Pianka index (Pianka 1973). An index value of zero stands for complete partitioning (plants producing nectar at different times of the day), while at unity, we would conclude no partitioning (plants producing nectar at the same times). We tested if the observed diel partitioning deviated from random expectation by assessing the proportion of times it was outside the 10,000 null expectations generated by the ROSARIO algorithm (Castro-Arellano et al. 2010).

We further assessed diel partitioning at the plant species level by comparing the diel partitioning of the pollinator niche between pairs of plant species that (i) co-flowered or did not co-flower, and (ii) did or did not share a least one interacting hummingbird species. We used a Wilcoxon rank test as implemented in R (R Core Team 2018) to assess if diel partitioning (the Pianka indices of each plant species pair) differed between the groups (co-flowering or not, sharing interaction partners or not).

Since we expected plant species with more similar flower traits to present a higher diel partitioning of the pollinator niche, we correlated dissimilarity between both anther height and hourly nectar production of plant species pairs visited by hummingbirds. We performed a Mantel test with 10,000 iterations (Mantel 1967). To this end, we built a morphological distance matrix of anther height among plant species to relate it to a second distance matrix of hourly nectar production among plant species. We used the vegan R-package (Oksanen et al. 2013) to perform the Mantel test.

## Results

### Plant-hummingbird visits

**Fig. 2:**
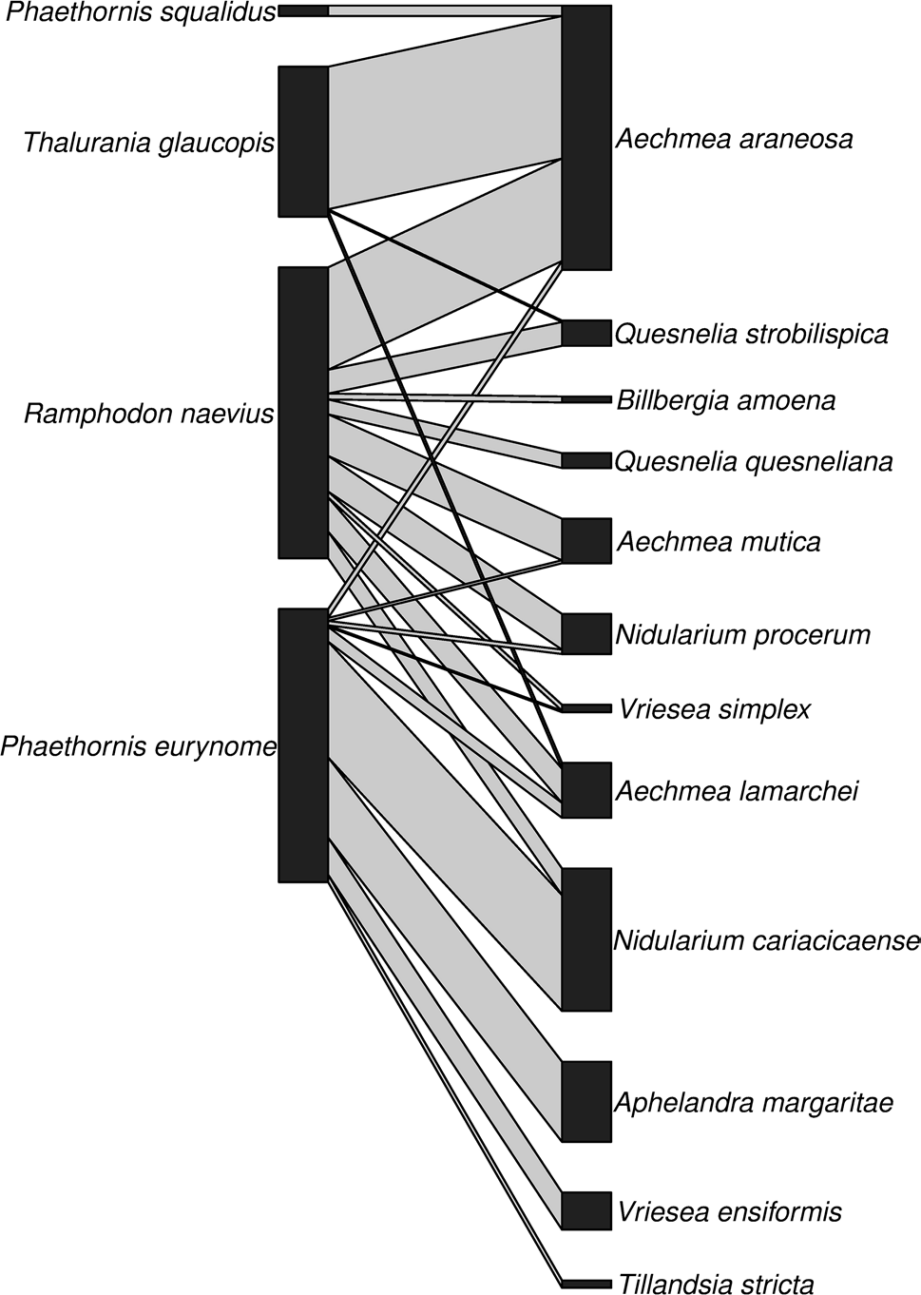
Hummingbird-plant mutualistic network recorded at the EBSL, southeastern Brazil. Hummingbirds are on the left side, and plants are on the right side of the network. Grey lines represent species interactions, and line thickness indicates the frequency of interactions

**Fig. 3:**
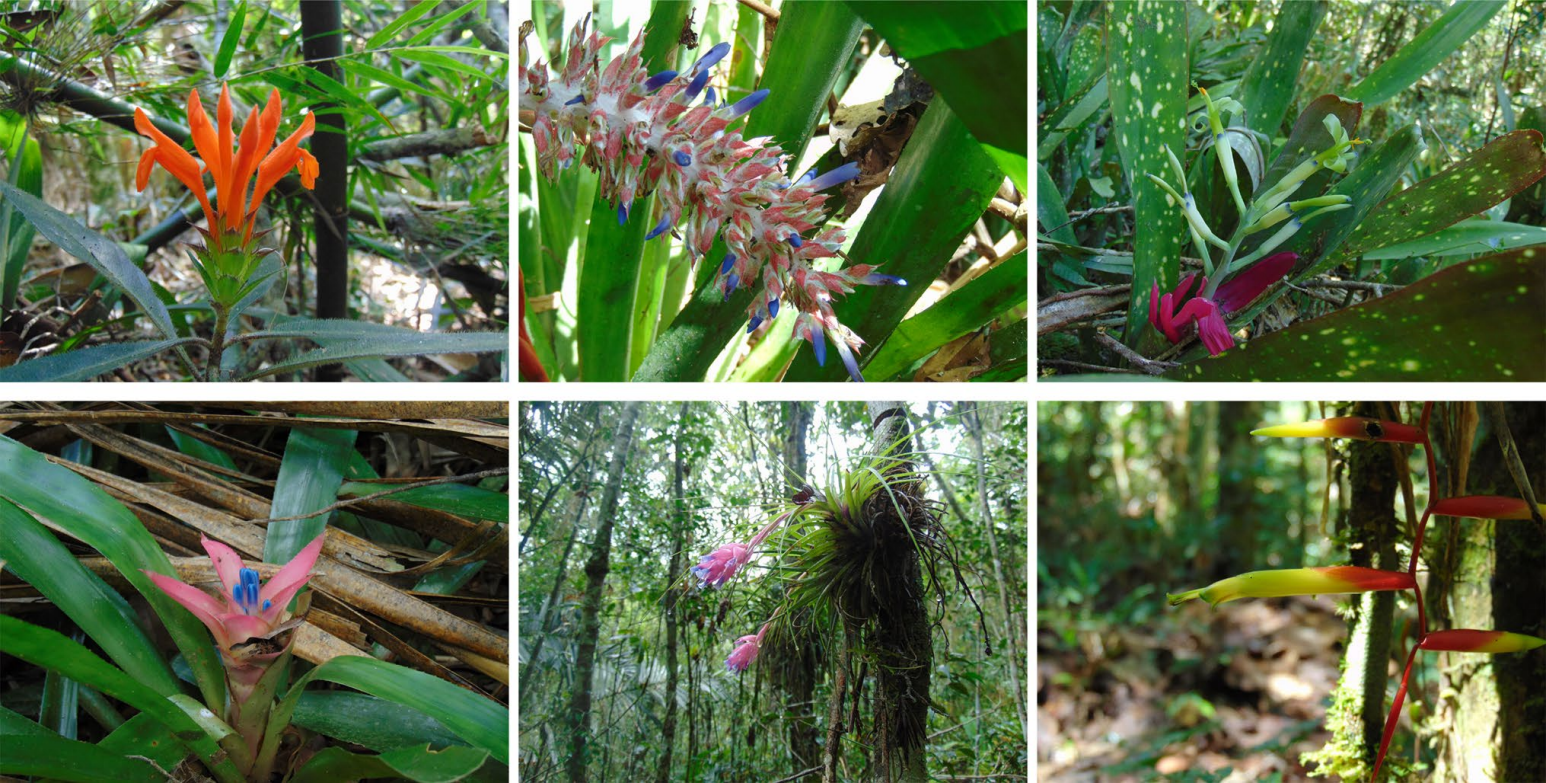
Some of the plant species visited by hummingbirds at the EBSL, southeastern Brazil. From left to right and from top to bottom; *Aphelandra margaritae*, *Aechmea mutica*, *Billbergia amoena*, *Nidularium cariacicaense*, *Tillandsia stricta* and *Vriesea simplex*

For eight months, from Nov-2018 to Jul-2019, we recorded 488 legitimate visits between four hummingbird species, *Phaethornis eurynome*, *Phaethornis squalidus*, *Ramphodon naevius*, and *Thalurania glaucopis*, and 12 plant species (Table 1, Fig. 2). Most of the plant species visited by hummingbirds belonged to Bromeliaceae (88%), followed by Acanthaceae (12%) (Fig. 3). Most hummingbird visits (56%) occurred between 06:00 and 10:00 h (Fig. 1). *Ramphodon naevius* made the highest number of visits (196) to nine bromeliad species; the most visited were *Aechmea araneosa* (35% visits) and *Aechmea mutica* (14% visits). *Phaethornis eurynome* made 184 visits to eight bromeliad species and one species from Acanthaceae. The most visited plants were *Nidularium cariacicaense* (42%) and *Aphelandra margaritae* (29%). *Thalurania glaucopis* made 101 visits to four bromeliad species, and most were on *A. araneosa* (95% visits). *Phaethornis squalidus* made seven visits on *A. araneosa*.

**Table 1:** Interactions between plant (rows) and hummingbird (columns) species recorded at the EBSL, southeastern Brazil. Numbers are total visits by each hummingbird species to each plant species during the study period

	*Aechmea araneosa*	*Aechmea lamarchei*	*Aechmea mutica*	*Aphelandra margaritae*	*Billbergia amoena*	*Nidularium cariacicaense*	*Nidularium procerum*	*Quesnelia quesneliana*	*Quesnelia strobilispica*	*Tillandsia stricta*	*Vriesea ensiformis*	*Vriesea simplex*
*Phaethornis eurynome*	6	10	2	54	0	78	3	0	0	5	25	1
*Phaethornis squalidus*	7	0	0	0	0	0	0	0	0	0	0	0
*Ramphodon naevius*	69	23	28	0	4	18	24	10	16	0	0	4
*Thalurania glaucopis*	96	4	0	0	0	0	0	0	1	0	0	0

### Flower abundance and traits

**Fig. 4:**
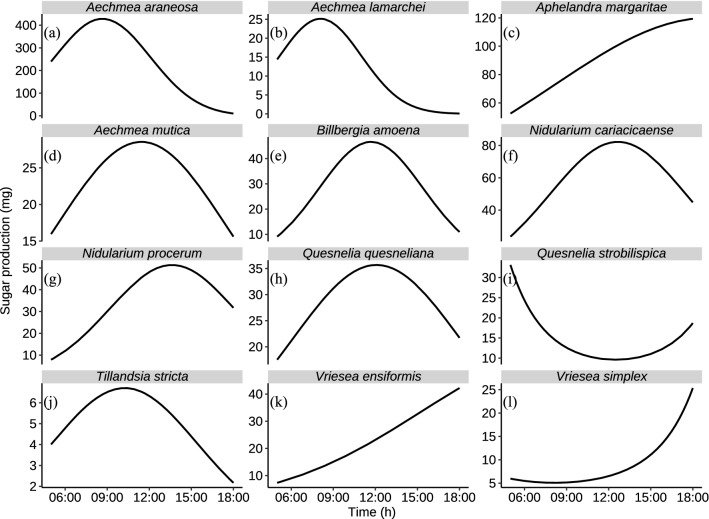
Hourly nectar production (mg sugar) for **a** *Aechmea araneosa*, **b** *Aechmea lamarchei*, **c** *Aechmea mutica*, **d** *Aphelandra margaritae*, **e** *Billbergia amoena*, **f** *Nidularium cariacicaense*, **g** *Nidularium procerum*, **h** *Quesnelia quesneliana*, **i** *Tillandsia stricta*, **j** *Vriesea ensiformis*, **k** *Vriesea simplex* and, **l** *Quesnelia strobilispica*. Each line represents the pattern of nectar production throughout the day of plant species visited by the hummingbird assemblage at the EBSL, southeastern Brazil

Across the study period, *A. araneosa* was the most abundant species with 103 flowers, while *Quesnelia quesneliana* was the least abundant with three flowers. Nectar production ranged from 0.02 mg day^−1^ of sugar in *Aechmea lamarchei* to 8.44 mg day^−1^ in *Vriesea ensiformis*. When we calculated the availability of the total resources (mg sugar x flowers abundance), *A. lamarchei* provided the least sugar (0.08 mg day^−1^) while *A. araneosa* provided the most (426.4 mg day^−1^, Fig. 4). *Aphelandra margaritae*, *V. ensiformis*, and *Vriesea simplex* increased their nectar production at 16:00 h. *Quesnelia strobilispica* showed an exceptional pattern in that it had no peak but rather a period of minimal nectar production between 10:00 and 15:00 h. The remaining plant species had a peak of nectar production between 07:00 and 13:00 h (Fig. 4). Anther height ranged from 0.67 cm (SD = 0.10, n = 5) in *Tillandsia stricta* to 7.67 cm (SD = 0.58, n = 5) in *V. simplex* (Table 2).

**Table 2:** Mean anther height (cm) and nectar production (mg sugar) equation of the plant species visited by the hummingbird assemblage at the EBSL, southeastern Brazil. In the equation, x = time and y = nectar production (NP) (mg of sugar)

Plant species	Anther height (cm)	Nectar production equation
*Aechmea araneosa*	1.76	$NP=\mathrm{exp}(5.086 +0.749time-0.043{x}^{2})$
*Aechmea lamarchei*	2.50	$NP=\mathrm{exp}\left(4.927 +0.943time -0.058{x}^{2}\right)$
*Aechmea mutica*	3.06	$NP=\mathrm{exp}\left(6.227 +0.320time -0.014{x}^{2}\right)$
*Aphelandra margaritae*	4.73	$NP=\mathrm{exp}\left(5.906 +0.157time -0.004{x}^{2}\right)$
*Billbergia amoena*	5.51	$NP=\mathrm{exp}\left(3.684 +0.852time -0.036{x}^{2}\right)$
*Nidularium cariacicaense*	4.84	$NP=\mathrm{exp}\left(4.409 +0.539time -0.021{x}^{2}\right)$
*Nidularium procerum*	4.11	$NP=\mathrm{exp}\left(3.343 +0.685time -0.025{x}^{2}\right)$
*Quesnelia quesneliana*	3.24	$NP=\mathrm{exp}\left(6.466 +0.342time -0.014{x}^{2}\right)$
*Quesnelia strobilispica*	4.95	$NP=\mathrm{exp}\left(10.661-0.549time+ 0.021{x}^{2}\right)$
*Tillandsia stricta*	0.67	$NP=\mathrm{exp}\left(4.362 +0.383time -0.018{x}^{2}\right)$
*Vriesea ensiformis*	5.56	$NP=\mathrm{exp}\left(6.183 +0.246time -0.004{x}^{2}\right)$
*Vriesea simplex*	7.67	$NP=\mathrm{exp}\left(8.235-0.267time+ 0.016{x}^{2}\right)$

### Hummingbirds niche partitioning

The hummingbird assemblage showed significant partitioning for trophic niche partitioning, indicating that hummingbird species foraged mostly on different plants (p = 0.01, Table 3). In contrast, for diel niche partitioning, hummingbirds showed less partitioning than expected by chance, indicating that hummingbirds foraged at the same times both in the morning (p = 0.96) and throughout the day (p = 1.00, Table 3).

**Table 3:** Niche partitioning patterns for hummingbirds and plants. Trophic niche partitioning evaluates whether the hummingbird assemblage foraged on distinct plant species. Diel niche partitioning evaluates whether hummingbird assemblage foraged on distinct hours throughout the day. Diel partitioning of the pollination niche evaluates whether the plant community partitioned pollinator visits throughout the day. The observed values (0 = complete partitioning, 1 = complete overlap) are the niche partitioning (Pianka index) calculated for the hummingbird assemblage and the plant community visited by hummingbirds at EBSL, southeastern Brazil. p values are one-tailed probabilities of finding partitioning patterns. p value was calculated as the proportion of randomizations that resulted in partitioning that is equal to or less than the observed partitioning

	Model	Observed	p value
Hummingbird assemblage (trophic niche partitioning)	ra3	0.50	0.0104*
Hummingbird assemblage (diel niche partitioning throughout the day)	ROSARIO	0.88	1.00
Hummingbird assemblage (diel niche partitioning throughout the morning)	ROSARIO	0.88	0.9691
Plant community (diel partitioning of the pollination niche throughout the day)	ROSARIO	0.85	0.3900

*Indicates a significant value for niche partitioning

### Plants niche partitioning

**Fig. 5:**
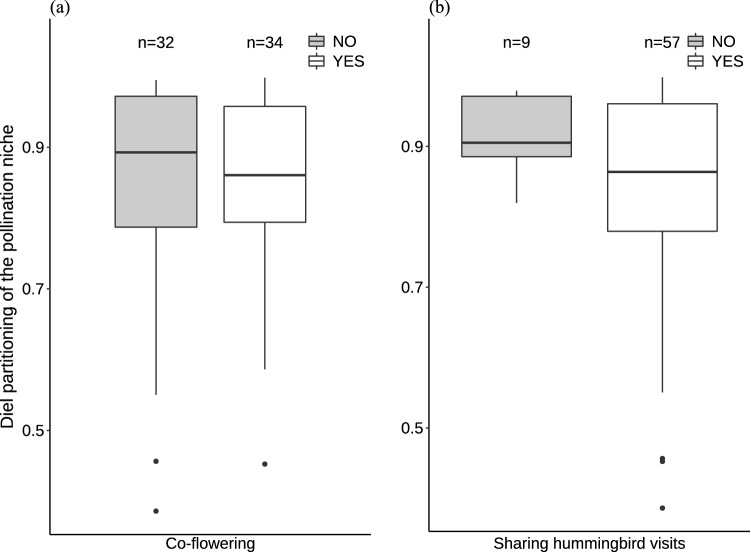
Diel partitioning of the pollination niche between plant species that **a** co-flowered (white) and did not coflower (grey) and, **b** shared (white) and did not share (grey) a hummingbird visit at the EBSL, southeastern Brazil. Partitioning values range from 0 = complete partitioning for pairs of plant species producing nectar at different times and thus, partitioning the pollination niche (pollinator visits) throughout the day, to 1 = complete overlap, for pairs of plant species producing nectar at similar times and thus, overlapping the pollination niche (pollinator visits) throughout the day. Boxplots show median values (thick lines), interquartile (the box), and confidence intervals (whiskers)

At the community level, diel partitioning of the pollinator niche did not differ from the null model expectation (p = 0.39, Table 3), indicating that plants did not partition the visits of pollinator species throughout the day. At the species level, there were 66 plant species pairs, of which 34 co-flowered at least once and 57 shared at least one hummingbird species. The diel partitioning of the pollinator niche did not vary when plant species co-flowered (one-tailed test, W = 572, p = 0.36, Fig. 5a) or shared at least one hummingbird species (one-tailed test, W = 319, p = 0.12, Fig. 5b). There was no correlation between anther height and nectar production dissimilarity (Mantel r = 0.08, p = 0.26).

## Discussion

Diel partitioning was not observed in either the hummingbird resource niche or plant-pollinator niche indicating that the very fine temporal niche partitioning throughout a day likely does not have a significant role in structuring the observed hummingbird-plant interaction network. However, we observed trophic niche partitioning in hummingbirds suggesting that competitive interactions, trait matching, or other behavior characteristics (such as trap-lining) result in each hummingbird species preferentially using a unique subset of plants. These results add to a growing literature that trophic niche partitioning is an important mechanism for structuring plant-hummingbird interaction networks and fostering the co-existence of multiple species within a single family (e.g., Feisinger and Colwell 1978; Maglianesi et al. 2015; Weinstein and Graham 2017; Sonne et al. 2019). In contrast, facilitation may be important in the studied plant communities because plants that co-flowered or shared pollinators produced nectar at similar times throughout the day (Moeller 2004; Bergamo et al. 2018). Our results emphasize that interacting trophic groups may have different strategies for maximizing fitness and that evaluating both sides of the network is critical to gaining a better understanding of the mechanisms underlying trophic interactions (Dehling et al. 2016).

Trophic niche partitioning among hummingbirds may be influenced by floral resource availability and morphology imposed by the floral traits. The EBSL tends to show a less marked flowering season (Varassin 2002) than other similar areas of the Atlantic forest (Martinelli 1997). For instance, we observed that different species in the family Bromeliaceae flowered continuously and offered nectar throughout the day, suggesting that the sequential flowering of bromeliads may strongly influence resource availability (Varassin 2002). Despite continuous flowering, hummingbirds partitioned floral resources by selecting a subset of plants for foraging, indicating that resources were limited, leading hummingbirds to compete for flowers. Some hummingbirds, such as *Phaethornis eurynome*, may select long-corolla flowers, e.g., *Nidularium cariacicaense* and *Aphelandra margaritae,* as they provide more nectar reward than short-corolla flowers (Ornelas et al. 2007; Maglianesi et al. 2015). Other hummingbirds, such as *Thalurania glaucopis*, foraged almost exclusively on short-corolla flowers, e.g., *Aechmea araneosa*. Our findings are consistent with previous studies that suggest trophic niche partitioning, resulting from competition and influenced by resource availability and trait-matching, as an important factor in structuring hummingbird communities (Graham et al. 2009, 2012; Parra et al. 2011) (Table 4).

**Table 4:** Floral morphological traits of some plant species visited by the hummingbird assemblage at the EBSL, southeastern Brazil. The length of the corolla tube, stigma and anther was measured from the base of the corolla to the tip of each of the aforementioned parts

Plant species	n^a^	Flower shape	Bract color	Calyx color	Corolla color	Corolla symetry^b^	Pollination syndrome^c^	Mean corolla tube length (cm)	Mean stigma length (cm)	Mean anther length (cm)
*Aechmea araneosa*	5	T	Red	Green	Yellow to orange	A	H	1.60	1.79	1.76
*Aechmea lamarchei*	5	T	Red	Green	Yellow	A	H	2.77	2.70	2.51
*Aechmea mutica*	6	T	Red	Pink	Light purple	A	H	2.39	3.06	3.06
*Aphelandra margaritae*	14	T	None	Green, red apex	Orange to red	Z	H	3.97	4.94	4.73
*Billbergia amoena*	6	T	Pink	Green	Light green	Z	H	4.99	6.71	5.51
*Nidularium cariacicaense*	7	T	Red	Green/red	Dark purple	A	H	5.20	4.69	4.84
*Nidularium procerum*	13	T	Red	Green	Dark purple	A	H	5.10	4.47	4.10
*Quesnelia quesneliana*	4	T	White, pink apex	Pink	White and purple	Z	H	3.69	3.16	3.25
*Quesnelia strobilispica*	5	T	Pink	Pink	White, blue apex	Z	H	5.07	5.02	4.95
*Tillandsia stricta*	5	T	Pink	Pink	Purple to blue	A	H	1.12	0.72	0.67
*Vriesea ensiformis*	7	T	Red	Red	Yellow	Z	H	5.02	5.52	5.56
*Vriesea simplex*	5	T	Red	Green to yellow	Yellow, green apex	Z	H	6.45	7.69	7.67

^a^Number of flowers measured

^b^Actinomorphic (A), Zygomorphic (Z)

^c^Hummingbird (H)

There are several explanations for the lack of diel niche partitioning among hummingbirds. First, niche partitioning occurred on a trophic rather than diel scale, i.e., hummingbirds visited mostly different plant species, therefore, diel partitioning was unnecessary. Second, the foraging behavior of hummingbirds differed across species. *Phaethornis eurynome* and *Ramphodon naevius* are high-reward trap-liner species (Feinsinger and Colwell 1978; Stiles and Freeman 1993) that exploit nectar-rich flowers dispersed in spatial clumps in the Atlantic forest (Sazima et al. 1995; Buzato et al. 2000), whereas *Thalurania glaucopis*, an aggressive territorial hummingbird, generally defends clumped rich flowers (Canela 2006; Missagia and Alves 2016). The potential dominance over some floral resources by *T. glaucopis* may have contributed to the lack of diel niche partitioning. Third, the hummingbirds we observed are closely related and may be evolutionarily constrained to being active simultaneously (Daan 1981; Kronfeld-Schor et al. 2001a, b, c; Kronfeld-Schor and Dayan 2003). As a result, hummingbirds have similar foraging times throughout the day leading to overlapping activity patterns due to niche conservatism (Wiens and Graham 2005; Losos 2008). These results do not signal competition at a fine temporal scale; on the contrary, niche partitioning mechanisms occur at a trophic scale.

The lack of diel partitioning of the pollinator niche in plants when they co-flowered or shared hummingbird visits suggests that positive rather than negative interactions may be structuring the plant community. The potential benefit of co-flowering and offering nectar at similar times is the joint attraction of pollinators (Moeller 2004) and increased visitation, which is likely to favor the fitness of plants, especially rare species (Feldman et al. 2004; Bergamo et al. 2020a, b). Interestingly, a recent study found a positive relationship between heterospecific pollen deposition and fruit set of hummingbird-pollinated plants suggesting that sharing both flowering times and pollinators may promote facilitation and reproductive benefits for plants (Lopes et al. 2022). Also, because hummingbird-pollinated plants tend to be pollen-limited (Wolowski et al. 2013), facilitation via co-flowering may help overcome pollen limitation by jointly attracting pollinators (Bergamo et al. 2022). The absence of a relationship between floral morphology and nectar production suggests that plants sharing hummingbird visits at the same time throughout the day may not always present fine adjustment in pollen placement traits (i.e., differences in anther height or position), a mechanism known to limit heterospecific pollination in facilitative interactions (Ruchisansakun et al. 2016; Bergamo et al. 2017). Besides that, the similarity of floral traits among species may increase the reproductive success of plants sharing their pollination niche (i.e., pollinators, flowering times, or diel nectar production) (Moeller 2004). The absence of diel partitioning of the pollination niche among plants suggests facilitative interactions mediated by morphological similarity within the studied community, as Bergamo et al. (2018) reported for many traits, including nectar content.

Our focus on fine-scale temporal patterns provided a more nuanced and detailed view of mechanisms influencing plant-hummingbird interactions and revealed that hummingbirds and plants are under different regimes of niche partitioning. Different regimes on both sides of the network (pollinator and plant) highlight the importance of considering different conservation strategies for hummingbirds and plants and their function in the ecosystem. For hummingbirds, a diverse set of species with different flower morphologies are likely required because trophic niche partitioning emerged as an important mechanism influencing community structure and hence will be important for the maintenance of hummingbird diversity. For plants, pollinator diversity and positive interactions (such as facilitation through pollinator sharing and co-flowering) may be essential to sustain plant reproduction and likely influence the establishment and persistence of plant communities (Sargent and Ackerly 2008). Studies such as this one, which evaluate both sides of a mutualistic network, help us to identify the conditions that could determine the outcome of biotic interactions (positive and negative) and, therefore, anticipate the different ecological effects of these interactions. Although there is a consensus that trophic partitioning is a decisive mechanism shaping diverse communities, diel niche partitioning should not be discounted because only a few studies have looked for it, and those that have found mixed results (Lara et al. 2009, 2011). Ideally, as studies of diel partitioning accumulate, it will be possible to determine when and under what conditions this mechanism may be important for structuring and maintaining diversity.

## Data Availability

The analyzed data will be available after acceptance in an appropriate repository.
